# HTLV-1 associated bronchiectasis among Indigenous Australians is associated with higher HTLV-1 proviral loads: Results of a prospective case-control study

**DOI:** 10.1186/1742-4690-11-S1-P28

**Published:** 2014-01-07

**Authors:** Lloyd Einsiedel, Olivier Cassar, Emma Goeman, Tim Spelman, Antoine Gessain

**Affiliations:** 1 Flinders University/Northern Territory Rural Clinical School, Alice Springs Hospital, Northern Territory, Australia; 2 Institut Pasteur, Unité d’Epidémiologie et Physiopathologie des Virus Oncogènes, Département de Virologie, Paris, France; 3 CNRS, UMR 3569, Paris, France; 4 Department of Paediatrics, Alice Springs Hospital, Northern Territory, Australia

## 

Infection with HTLV-1 is associated with bronchiectasis in Indigenous Australians. Previous studies have not determined HTLV-1 proviral loads (pvl), which predict risk of other HTLV-1 associated inflammatory diseases, such as HTLV-1 associated myelopathy. Thirty-six Indigenous adults admitted to Alice Springs Hospital, June 2008 to December 2009, with radiologically confirmed bronchiectasis, but no other HTLV-1 related disease, were prospectively recruited and matched by age and sex to 36 controls of the same ethno-geographic origin. Case notes were reviewed from date of birth or first admission to date of recruitment. HTLV-1 subtype C pvl were determined at the Pasteur Institute, Paris. HTLV-1 infection was more common among cases (25/36; 69.4%) than their controls (15/36; 41.7%)(p<0.018). Two cases were admitted in childhood with probable infective dermatitis and two with recurrent strongyloidiasis unresponsive to treatment with thiabendazole. In adulthood, cases (25/36; 69%) were more likely than controls (5/25; 20%) to have positive or borderline strongyloides serology (p<0.001). The mean HTLV-1 pvl (±SEM) was significantly higher for cases (0.83±0.21% PBMC) than controls (0.12±0.05% PBMC)(p=0.016) and was nearly17 fold-higher compared to controls in which bronchiectasis was excluded by HRCT (0.05±0.02% PBMC; p=0.006). Twelve cases (33.3%) and 5 controls (13.9%) died during 3 years of follow-up (p<0.052). Cases died at a younger age (cases, 49±15; controls, 60±13)(p=0.15). In both groups HTLV-1 infected patients were more likely to die: cases, 11/12 (92%); controls, 4/5 (80%). HTLV-1-associated bronchiectasis is associated with higher HTLV-1 pvl suggesting that this condition results from an HTLV-1 driven inflammatory process.

